# Effects of depressive symptoms on neuronal processing of social evaluative feedback and subsequent changes in expectations and self-view

**DOI:** 10.1017/S0033291725102511

**Published:** 2025-11-26

**Authors:** Hanne Helming, Antje Peters, Franka Hüttenhein, Robert Moeck, Thomas Straube, Sebastian Schindler

**Affiliations:** 1 Institute of Medical Psychology and Systems Neuroscience, University of Münster, Muenster, Germany; 2 Otto Creutzfeldt Center for Cognitive and Behavioral Neuroscience, University of Muenster, Muenster, Germany

**Keywords:** depression, EEG/ERPs, self-view updating, social evaluative feedback

## Abstract

**Background:**

Social interaction is a primary aspect of communicating how others judge us. It allows us to update ourselves and our expectations about others. While humans generally exhibit self-related positive biases in their updating behavior, theoretical accounts propose that this biased processing is attenuated, absent, or negatively biased in participants with depressive symptoms. The process of aligning and integrating social evaluative feedback in realistic interaction scenarios that would test this assumption is, however, lacking. We provide an event-related potential (ERP) study that combines neuronal (feedback-related negativity [FRN] and late positive potential [LPP]) and behavioral measures of evaluative feedback processing and updating behavior.

**Methods:**

We selected healthy adults (*N* = 62) with depression scores spanning a range of low to high values, as measured by the Beck Depression Inventory (BDI). Participants received feedback from supposed experts and peer senders, with the feedback being manipulated to be worse, congruent, or better than the participants’ self-ratings.

**Results:**

Participants with higher depression scores exhibited more negative initial self-ratings and developed a more negative feedback expectation across the experiment. In addition, we found that higher depression scores led to more negative updating toward worse expert feedback and less positive updating after better peer feedback. Concerning ERPs, unexpected but not self-incongruent feedback increased the FRN, while both types of incongruence increased the LPP. Finally, BDI scores correlated with LPP amplitudes for all feedback.

**Conclusions:**

The results contribute to a deeper understanding of how individuals process and integrate social evaluative feedback and its relation to depressive symptoms.

## Background

Developing a self-view over the lifespan is influenced by how we see ourselves and how we think others see us (Andersen et al., 1997). This requires communication and the exchange of such views in social interaction. Theoretical accounts propose that the self is shaped by integrating evaluations from significant others (Lundgren, 2004; Mead, 1934), and research has shown that the integration is influenced by both the sender and receiver characteristics (Collins & Stukas, 2006; Falk & Scholz, 2018). Positive social evaluations fulfill the psychological need to belong (Baumeister & Leary, 1995), whereas negative social evaluations increase the risk of isolation, depression, anxiety, and early death (Cacioppo et al., 2006). Specifically, dating back to Beck, participants with depression are supposed to exhibit negative perceptions of the self, the world, and the future (Beck, 1979). It remains unclear how characteristics of depressive symptoms impact responses toward social evaluative feedback concerning the changes in expectations, changes of self-view, and the underlying neuronal responses.

Humans have been described as being overly optimistic about their future and, when informed about risks, selectively updating beliefs more in response to new positive information (Garrett & Sharot, 2017; Kuzmanovic et al., 2015; Sharot et al., 2011, 2012). People also view themselves as superior to their peers and remember positive feedback more strongly (Hepper et al., 2011). Unsurprisingly, integrating social evaluative feedback is biased, showing more adaptation toward desirable (i.e. positive) feedback (Elder, Davis, & Hughes, 2022; Korn, Prehn, Park, Walter, & Heekeren, 2012). Positive social evaluative feedback also affects recognition memory with an overestimation of the occurrence of past positive feedback (Schindler, Höhner, Moeck, Bruchmann, & Straube, 2021). However, the biased processing in self-related risk updating is inhibited or even absent in depressed individuals. Their updating of beliefs seems equally for good and bad news (Strunk et al., 2006; Sharot, 2011). Note that it is unclear whether negative belief updating leads to pessimism and low mood or if low mood impairs belief updating about the future (Sharot, 2011). Depressive symptoms have been related to less learning from positive information (Kube, Schwarting, Rozenkrantz, Glombiewski, & Rief, 2020; Pinquart et al., 2021), and it has been suggested that cognitive immunization hinders changing negative beliefs in individuals with elevated depressive symptoms (Kube, 2023; Kube & Glombiewski, 2021, 2022).

Processing of social evaluative feedback can be examined using the high temporal resolution of event-related potentials (ERPs) from the EEG. Two ERP components are of high importance concerning the processing and integration of feedback: the feedback-related negativity (FRN) and the late positive potential (LPP). The FRN is detected during mid-latency processing stages (160–330 ms), over fronto-central sensors and reasoned to originate from the Anterior Cingulate Cortex (Becker, Nitsch, Miltner, & Straube, 2014; Hauser et al., 2014), and increased for generally unexpected or unexpected negative evaluative feedback (Peters et al., 2024). A different view emerged on the role of this component, as reflecting the variability of positive deflections to positive outcomes (Bernat, Nelson, & Baskin-Sommers, 2015; Foti, Weinberg, Bernat, & Proudfit, 2015). The LPP emerges from approximately 400 ms onward after stimulus appearance, often showing a broad central topography (see Hajcak, Dunning, & Foti, 2009; Schupp, Flaisch, Stockburger, & Junghöfer, 2006), and is hypothesized to reflect the activation of broad and distributed brain regions (Liu, Huang, McGinnis-Deweese, Keil, & Ding, 2012; Sabatinelli et al., 2014). The LPP is related to elaborate stimulus processing, including stimulus evaluation, self-referential processing, and information integration (Dolcos & Cabeza, 2002; Hajcak, MacNamara, & Olvet, 2010). Findings on social evaluative feedback show that more relevant incongruent feedback reliably increases the LPP (Schindler et al., 2021; for review, see Peters et al., 2024). Only a few studies on social evaluative feedback processing examined relations with depressive symptoms, two of them reporting relationships between depression and less positive amplitudes toward acceptance feedback in the FRN time window (Pegg et al., 2019; Pegg, Arfer, & Kujawa, 2021), or absent differential LPP responses between acceptance versus rejection feedback (Xie et al., 2022). However, the few findings are based on different designs and could not relate ERPs to updating behavior.

To understand the processing, integration, and updating of social evaluative feedback and modulations by sender and receiver characteristics, we manipulated social evaluative feedback regarding the sender’s feedback behavior and expertise in selected participants with varying severity of self-reported depression scores. We expected participants to change feedback expectations according to the overall sender behavior but would selectively change self-view ratings according to feedback being better than their self-view ratings. In both cases (i.e. expectations and self-rating updates), we expected that participants with higher depressive scores should show an attenuated positive integration of the feedback. Finally, we expected differences between feedback incongruent and congruent with the self-view and feedback expectations on the FRN and LPP components and tested previously reported relationships between depressive symptoms and ERPs.

## Methods

### Participants

A sample of 70 participants was recruited in Münster, Germany. We pre-screened participants according to their self-reported depressive scores and invited participants across the range of BDI scores (25 percentile = 3; median = 6; 75 percentile = 12; *Min* = 0, *Max* = 33). Eight participants were excluded due to not meeting the inclusion criteria, not completing the participation, or having extensive artifacts in the EEG data. The final sample (*N* = 62, 19 males, 43 females) consisted of native-level German speakers (mean age = 23, *SD* = 3.10). All participants were right-handed, had normal or corrected-to-normal vision, and based on their self-report, had no previous or current diagnosed neurological or psychiatric disorders. All participants provided written informed consent and received 12 Euros per hour of participation or three course credit points (psychology course credit system) for psychology students. The study was approved by the Deutsche Gesellschaft für Psychologie ethics committee.

### Material and stimuli

We assigned 180 adjectives to four word lists (see Table 1). Adjectives were pre-rated using the self-assessment manikins (Bradley & Lang, 1994) for valence, arousal, concreteness, and self-relevance of personality evaluations. Linguistic properties were matched using the dlex database (Heister et al., 2011) with counterbalanced list assignments to senders across participants. Participants completed the German versions of the BDI (Beck et al., 2009).

**Table 1.** Comparison of the four word lists

Variable	List 1 (N = 45)	List 2 (N = 45)	List 3 (N = 45)	List 4 (N = 45)	*F*-value (3,176)	*p*-value
Valence	5.21 (2.26)	5.24 (2.33)	5.35 (2.36)	5.43 (2.29)	0.08	.969
Arousal	3.99 (0.66)	4.07 (0.74)	3.93 (0.78)	4.13 (0.71)	0.71	.550
Self-relevance	5.95 (0.91)	6.09 (1.00)	6.15 (0.98)	6.24 (0.90)	0.13	.942
Concreteness	5.51 (1.11)	5.51 (1.14)	5.48 (1.23)	5.62 (1.24)	0.71	.551
Word length	9.91 (2.00)	9.96 (1.87)	9.96 (1.71)	9.71 (1.84)	0.18	.912
Word frequency	270 (368)	262 (307)	255 (348)	247 (363)	0.04	.991
Regularity	107 (240)	107 (173)	101 (208)	135 (255)	0.23	.877

*Note:* Standard deviations appear in parentheses below the means. Valence = 1 highly negative, 5 = neutral, 9 = highly positive; Arousal 1 = very low, 9 = very high; Concreteness 1 = very concrete, 9 = very abstract. Word frequency is depicted per million.

### Procedure

**Figure 1.**
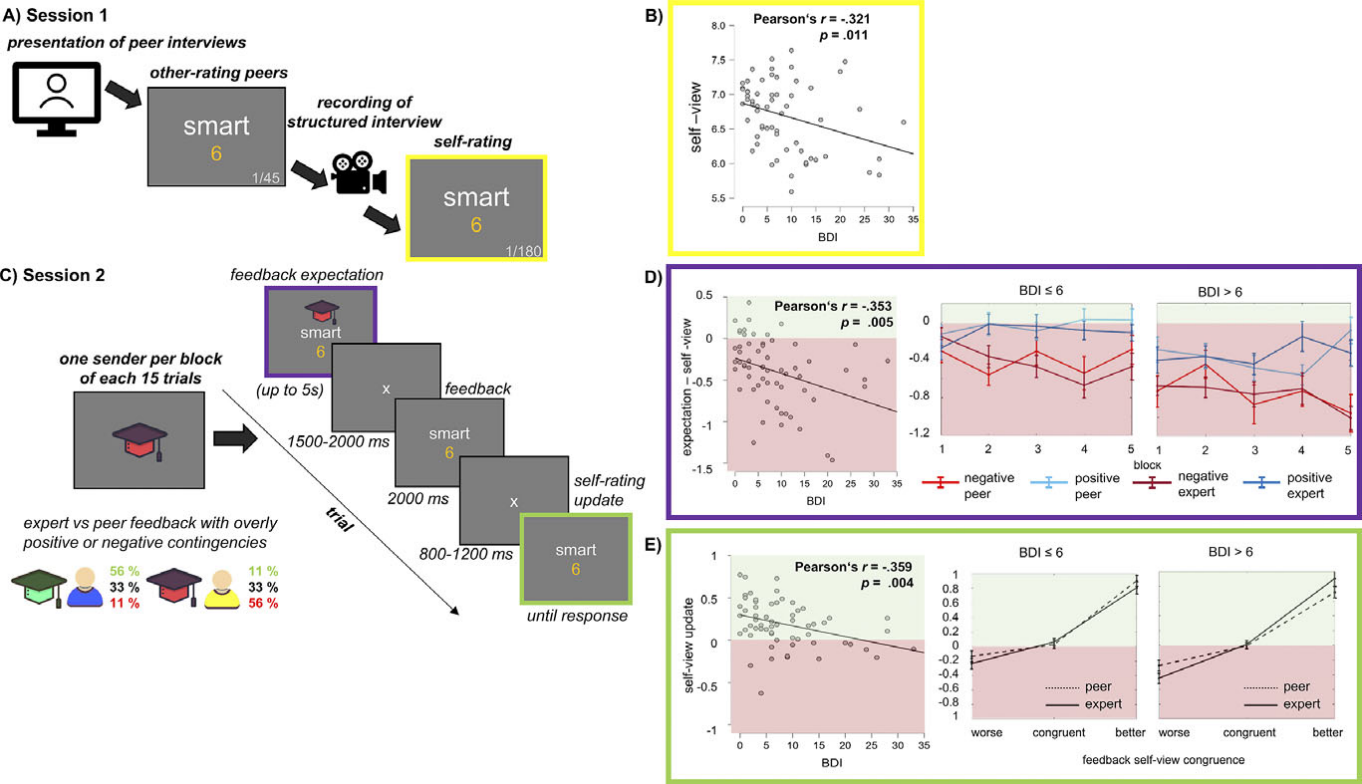
Schematic experimental procedures and behavioral results. (a) Session 1 recorded individual self-ratings. (b) Correlations between the BDI and the valence-inverted average initial self-rating (scale from 1 = most negative to 9 = most positive). (c) Session two presented feedback from four putative senders, two being introduced as experts and two as peers. Each sender provided alternating feedback in blocks of 15 trials. Participants had to indicate their feedback expectations first before being presented with the feedback, and subsequently, they were required to re-rate their respective traits. (d) Correlations between the BDI and the average difference between the self-rating and feedback expectations, next to the development of expectation per sender. (e) Correlations between the BDI and the average self-view updating values (−1 = negative to +1 = positive), next to the average updating per feedback type and sender. For illustrative purposes, the median split for participants with low and high BDI scores is shown in D and E.

The study took place at the Institute of Medical Psychology and Systems Neuroscience. Participants visited the laboratory for two appointments with a seven-day interval. The first appointment (see Figure 1a) served to assess the self-view and to create a credible feedback situation. After giving informed consent, participants watched videos of two supposed peers participating in the study and provided feedback by rating 45 adjectives on a scale from 1 (not applicable) to 9 (fully applicable). Afterward, participants introduced themselves in a 3-minute video, answering the same guideline questions using the previously validated structured interaction protocols that ask participants about their personality, strengths, and weaknesses (Schindler et al., 2015; Schindler, Miller, & Kissler, 2019). This video was supposedly the basis for the evaluation they would receive 1 week later. Finally, participants indicated their self-view ratings on all 180 adjectives using the same scales and filled in the BDI and demographic questionnaires (see Section “*Material and stimuli*”).

At the second appointment (see Figure 1c), the participants received feedback from four different senders while EEG data were recorded. The senders, consisting of two peers and two experts, were represented by a symbol and shortly introduced by a text. The senders were randomly assigned to one of the four lists of adjectives and thus provided feedback on 45 adjectives. The senders varied blockwise with 15 adjectives per block, and each block started with the sender symbol (see Figure 1c). The experiment consisted of a demo and practice trial before data collection and the 180 trials with two self-paced breaks. In each trial, after a fixation cross for 1500–2000 ms, an adjective with the symbol of the sender was presented, and participants had to indicate their expected feedback on a scale from one to nine. Then, a fixation cross of 1500–2000 ms was presented, followed by the feedback for 2000 ms. The feedback was manipulated, having one expert and one peer providing overly positive feedback (56% better, 33% congruent, 11% worse; see Figure 1c), while the other two provided overly negative feedback (11% better, 33% congruent, 56% worse, see Figure 1c). Thus, the feedback received by the participant consisted of 60 incongruent worse, 60 self-congruent, and 60 incongruent better evaluations, based on the initial self-rating indicated. The deviant feedback varied from one to three points from the indicated self-view. After another fixation cross for 800 to 1200 ms, the participants had to rate themselves again on the adjective. After the experimental trials, participants were debriefed about the nature of the study, and the feedback was manipulated, so no video of them was taken. After the experiment, 14 participants indicated doubts about the veridicality of the feedback upon request.

### EEG recording and preprocessing

EEG data were recorded from 64 BioSemi active electrodes using BioSemi’s ActiView software (version 8.11; www.biosemi.com). Additionally, four external electrodes measured horizontal and vertical eye movements. A Common Mode Sense active electrode (CMS) and a driven right Leg passive electrode (DLR) were used as ground electrodes. The offline data was preprocessed with BESA software (version 6.0; www.besa.de). The automatic eye-artifact correction was used to correct artifacts caused by eye movement, and low-quality channels were interpolated. The data were referenced to the average reference and filtered with a 0.1 Hz high-pass forward filter (6 dB/oct) and a 30 Hz low-pass zero-phase filter (24 dB/oct), segmented in epochs from 200 ms before feedback onset to 1500 ms after stimulus presentation with a baseline correction from 200 ms before the stimulus. For ERP analyses, we examined the FRN (166–326 ms) and the LPP (450–850 ms) components at the moment of feedback presentation. We identified and averaged mean amplitudes for the FRN over a frontocentral cluster (two electrodes: FCz, Cz) and the LPP over a fronto-central cluster (nine electrodes: F1, Fz, F2, FC1, FCz, FC2, C1, Cz, C2).

### Statistical analysis

For all analyses, adjectives with a negative valence, the rating r was inverted (r’ = 10 − r), so high values correspond to high positive ratings. Statistical analyses were performed using JASP (https://jasp-stats.org/ JASP Team, 2023). For behavioral effects, we tested feedback expectation ratings over time and self-view updating according to the feedback type and sender behavior. For expectation ratings, we averaged the expectation values within each trial across participants and five consecutive blocks, each containing nine trials per sender. We calculated a repeated-measures ANOVA with the factors sender expertise (two levels: expert vs. peer), sender attitude (two levels: negative vs. positive), and block (five levels: ‘Block 1’, ‘Block 2’, ‘Block 3’, ‘Block 4’, and ‘Block 5’). Concerning self-view updates, the update values were calculated based on differences between the initial rating and the re-evaluation after receiving feedback (t2 r’ – t1 r’), where negative values index negative changes and positive values index positive changes. Here, a repeated-measures ANOVA with the factor’s sender expertise (two levels: ‘peer’ vs. ‘expert’) and feedback self-view congruence (three levels: ‘congruent’ vs. ‘worse’ vs. ‘better’) was calculated. We correlated BDI scores with average self-view ratings at t1, with the average difference between the expectation and self-rating, and with the specific self-view updating differences, using two-sided Pearson correlations. For ERP analyses, we performed the same repeated-measures ANOVA with the factor’s sender expertise (two levels: ‘expert’ vs. ‘peer’) and feedback congruence to the self-view (three levels: ‘better’ vs. ‘congruent’ vs. ‘worse’) for FRN and LPP mean amplitudes and correlated ERP amplitudes with BDI scores for reported relationships in the literature (Pegg et al., 2019, 2021; Xie et al., 2022). Additionally, we run ANOVAs using the participants’ feedback prediction with the factor’s sender expertise (two levels: ‘expert’ vs. ‘peer’) and feedback expectedness (three levels: ‘better’ vs. ‘expected’ vs. ‘worse’). For all repeated-measures ANOVAs, p-values and effect sizes were corrected according to Greenhouse–Geisser whenever the Mauchly test violated the assumption of sphericity. For readability, the original degrees of freedom are reported. Post-hoc comparisons were corrected using the Bonferroni–Holm procedure. Partial eta-squared (η_P_^2^) and Cohen’s d were estimated to describe effect sizes (Cohen, 2013).

## Results

### Behavioral data

#### Expectation

Concerning expectation ratings, there was no main effect of sender expertise (*F*_(1,61)_ = 0.43, *p* = .514, η_P_^2^ = .007), while a significant main effect for the factor sender attitude (*F*_(1,61)_ = 74.19, *p* < .001, η_P_^2^ = .549) was found. Post-hoc comparisons revealed that feedback expectations were more positive for the positive than for the negative senders (*t* = 8.61, *p*_holm_ < .001, Cohen’s d = 0.520). There was no main effect of the block (*F*_(4,244)_ = 2.524, *p* = .084, η_P_^2^ = .040). We observed no significant interaction between sender attitude and sender expertise and block (*F*_(4,244)_ = 1.06, *p* = .379, η_P_^2^ = .017), between sender expertise and sender attitude (*F*_(1,61)_ = 0.01, *p* = .931, η_P_^2^ < .001), but a significant interaction between sender attitude and block (*F*_(4,244)_ = 3.19, *p* = .014, η_P_^2^ = .050). While positive and negative senders did not differ significantly in the first block (*t* = 2.25, *p*_holm_ = .477, Cohen’s d = 0.259), they did so in the second (*t* = 3.95, *p*_holm_ = .003, Cohen’s d = 0.454), third (*t* = 4.07, *p*_holm_ = .002, Cohen’s d = 0.468), fourth (*t* = 5.70, *p*_holm_ < .001, Cohen’s d = 0.656), and fifth blocks (*t* = 6.64, *p*_holm_ < .001, Cohen’s d = 0.763). There was no significant three-way interaction between sender expertise, sender attitude, and block (*F*_(1,61)_ = 0.98, *p* = .420, η_P_^2^ = .016). Concerning BDI scores, correlations accounted for initially more negative self-view (initial self-views displayed in Figure 1b; Pearson *r* = −.321, *p =* .011) by calculating the difference between expectations and the initial self-rating. Although we controlled for the initially more negative self-view of participants with higher BDI scores, participants with increasing BDI scores showed increasingly negative expectation ratings across the experiment (see Figure 1d; Pearson *r* = −.353, *p =* .005).

#### Self-view update

Self-view updating describes the change in the self-view between the first session and the second session (t1 r’ – t2 r’; see Figure 1e). The ANOVA revealed no main effect of sender expertise (*F*_(1,61)_ = 0.90, *p* = .345, η_P_^2^ < .001), a main effect of feedback congruence with the self-view (*F*_(2,120)_ = 232.83, *p* < .001, η_P_^2^ = .792), and the interaction of sender expertise and feedback congruence (*F*_(2,120)_ = 5.67, *p* = .004, η_P_^2^ = .085). Post-hoc analyses showed for the main effect of feedback congruence with the self-view that participants exhibited a significantly more positive self-view updating after receiving better feedback compared to congruent feedback to the self-view (*t*_(59)_ = 15.297, *p*_holm_ < .001, Cohen’s d = 2.038). Participants exhibited more negative updating after receiving worse feedback than the self-view compared to congruent feedback (*t*_(59)_ = −5.533, *p*_holm_ < .001, Cohen’s d = −0.737) and also compared to better feedback than the self-view (*t*_(59)_ = −20.83, *p*_holm_ < .001, Cohen’s d = −2.775). Concerning the interaction of sender expertise and feedback congruence with the self-view, updating did not differ between the senders after congruent feedback (*t*_(59)_ = 0.63, *p*_holm_ = .614, Cohen’s d = 0.065), and after better feedback than the self-view(*t*_(59)_ = 1.02, *p*_holm_ = .614, Cohen’s d = 0.105), while the expert sender led to more negative updating after worse feedback than the self-view(*t*_(59)_ = −3.30, *p*_holm_ = .004, Cohen’s d = −0.338). Again, correlational analysis with the BDI score for each participant revealed significant correlations in the case of the self-view updating aggregated over all conditions (see Figure 1e; Pearson *r* = −.359, *p =* .004). Following the significant sender-by-feedback interactions, correlations of higher BDI scores showed less positive updating after positive peer feedback (worse: Pearson *r* = −.209, *p =* .104; congruent: Pearson *r* = −.240, *p =* .060; better: Pearson *r* = −.413, *p <* .001) and more negative updating after worse expert feedback (worse: Pearson *r* = −.351, *p =* .005; congruent: Pearson *r* = −.193, *p =* .132; better: Pearson *r* = −.107, *p =* .406).

### ERPs results

#### FRN

**Figure 2.**
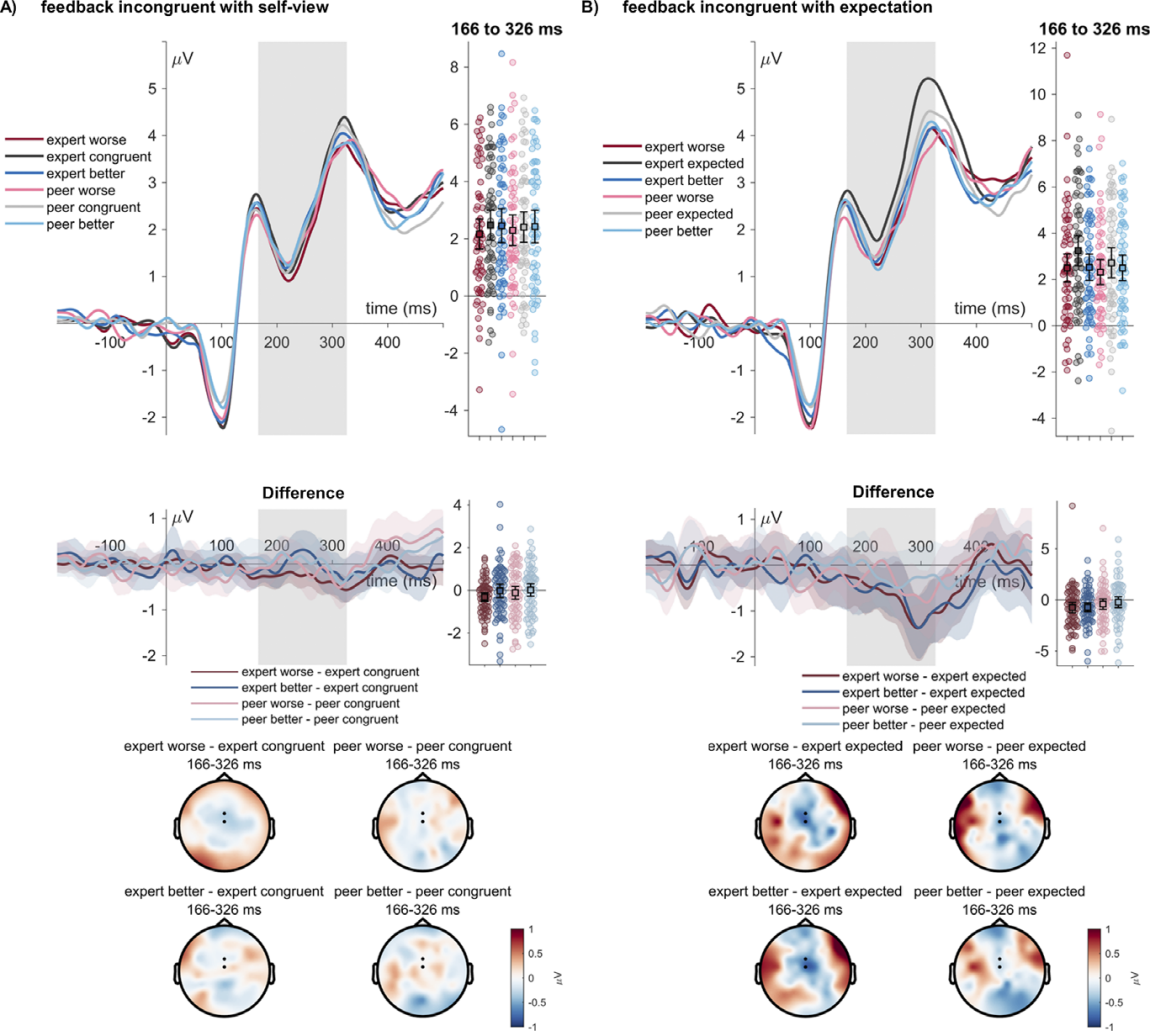
FRN effects of feedback. (a) Feedback incongruence with the intial self view ratings. (b) feedback incongruence with expectation ratings in the current trial. ERP waveforms show the time course for worse (red/pink), congruent (dark/light gray), and better feedback (dark/light blue lines) for the ‘peer’ and ‘expert’ senders. Error bars show 95% confidence intervals. Difference plots contain 95% bootstrap confidence intervals of intra-individual differences. Scalp topographies below depict the amplitude differences for the worse/better feedback and the congruent/expected feedback.

We examined event-related potentials to the feedback. Concerning the FRN, we observed for feedback relative to the self-view, no significant main effect of sender expertise (*F*_(1,61)_ = 0.02, *p* = .893, η_P_^2^ < .001), self-view congruence (*F*_(2,122)_ = 2.38, *p* = .097, η_P_^2^ = .037; see Figure 2a), and no interaction between sender expertise and self-view congruence (*F*_(2,122)*_ = 0.53, *p* = .572, η_P_^2^ = .009). Concerning BDI relationships with FRN amplitudes, we did not observe correlations between BDI scores and better, congruent, or worse feedback (worse: Pearson *r* = .114, *p =* .380; congruent: Pearson *r* = .132, *p =* .307; better: Pearson *r* = .105, *p =* .418).

Concerning feedback expectations, there was no main effect of the sender expertise (*F*_(1,61)_ = 3.96, *p* = .051, η_P_^2^ = .061), a main effect of the expectation (*F*_(2,122)*_ = 6.81, *p* = .003, η_P_^2^ = .100; see Figure 2b), and no interaction between sender and expectation (*F*_(2,122)_ = 1.10, *p* = .346, η_P_^2^ = .017). Post-hoc tests showed a significantly increased FRN for worse feedback than for expected feedback (*t*_(61)_ = −3.46, *p*_holm_ = .002, Cohen’s d = −0.239) and for better feedback than for expected feedback (*t*_(61)_ = − 2.84, *p*_holm_ = .011, Cohen’s d = −0.195). There was no difference between worse and better feedback (*t*_(61)_ = −0.63, *p*_holm_ = .532, Cohen’s d = −0.043).

#### LPP

**Figure 3.**
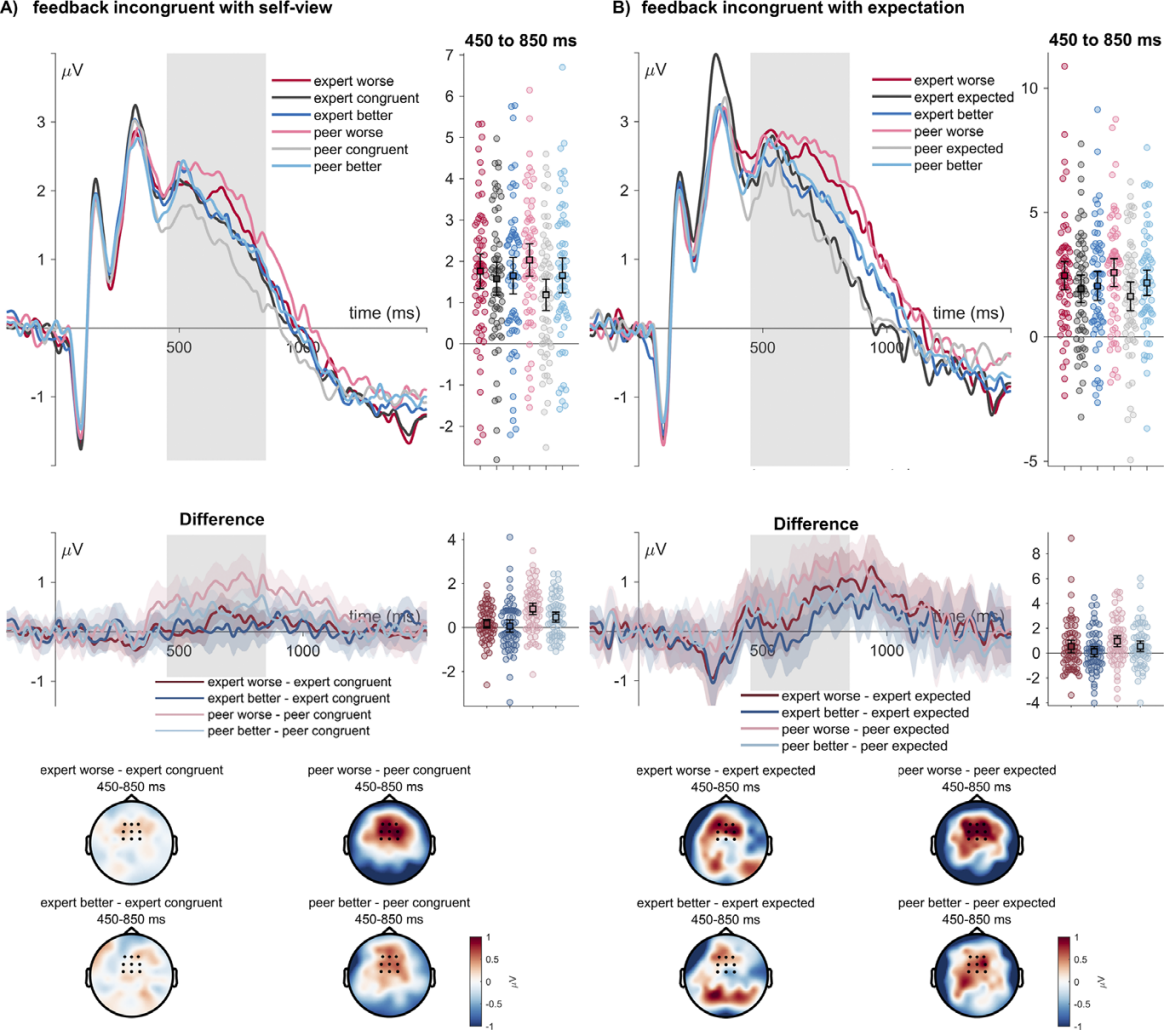
LPP effects of feedback. (a) Feedback incongruence with the intial self view ratings. (b) feedback incongruence with expectation ratings in the current trial.ERP waveforms show the time course for worse (red/pink), congruent (dark/light gray), and better feedback (dark/light blue lines) for the ‘peer’ and ‘expert’ senders. Error bars show 95% confidence intervals. Difference plots contain 95% bootstrap confidence intervals of intra-individual differences. Scalp topographies below depict the amplitude differences for the worse/better feedback and the congruent/expected feedback.

For the LPP, concerning feedback congruence with the self-view, there was no main effect of the sender (*F*_(1,61)_ = 0.16, *p* = .690, η_P_^2^ = .003), a main effect of the feedback congruence with the self-view (*F*_(2,122)_ = 13.91, *p* < .001, η_P_^2^ = .186; see Figure 3a), and an interaction between sender expertise and self-view congruence (*F*_(2,122)*_ = 7.92, *p* = .001, η_P_^2^ = .115). Post-hoc tests showed a larger LPP for worse feedback than for self-congruent feedback (*t*_(61)_ = 5.27, *p*_holm_ < .001, Cohen’s d = 0.309), and for worse than better feedback (*t*_(61)_ = 2.49, *p*_holm_ = .014, Cohen’s d = 0.146). Further, better feedback elicited a larger LPP than for self-congruent feedback (*t*_(61)_ = 2.79, *p*_holm_ = .012, Cohen’s d = 0.163). Concerning the interaction within the expert sender, worse and self-congruent feedback (*t*_(61)_ = 1.38, *p*_holm_ = 1.00, Cohen’s d = 0.107), better and self-congruent feedback (*t*_(61)_ = 0.54, *p*_holm_ = 1.00, Cohen’s d = 0.042), and worse and better feedback did not differ from each other (*t*_(61)_ = 0.85, *p*_holm_ = 1.00, Cohen’s d = 0.066). Within the peer sender, worse feedback (*t*_(61)_ = 6.57, *p*_holm_ < .001, Cohen’s d = 0.511) and better feedback elicited a larger LPP than self-congruent feedback (*t*_(61)_ = 3.67, *p*_holm_ = .004, Cohen’s d = 0.285). Worse feedback also led to a larger LPP than better feedback (*t*_(61)_ = 2.90, *p*_holm_ = .038, Cohen’s d = 0.226).

Concerning feedback expectations, there was no main effect of the sender expertise (*F*_(1,61)_ = 0.02, *p* = .878, η_P_^2^ < .001; see Figure 3b), a main effect of expectation (*F*_(2,122)_ = 9.85, *p* < .001, η_P_^2^ = .139), and no interaction between sender expertise and expectation (*F*_(2,122)*_ = 1.59, *p* = .213, η_P_^2^ = .025). Post-hoc tests showed a larger LPP for worse than for expected feedback (*t*_(61)_ = 4.43, *p*_holm_ < .001, Cohen’s d = 0.329), and between worse and better feedback (*t*_(61)_ = 2.47, *p*_holm_ = .030, Cohen’s d = 0.183), not being significantly different between better and excepted feedback (*t*_(61)_ = 1.97, *p*_holm_ = .052, Cohen’s d = 0.146).

**Figure 4.**
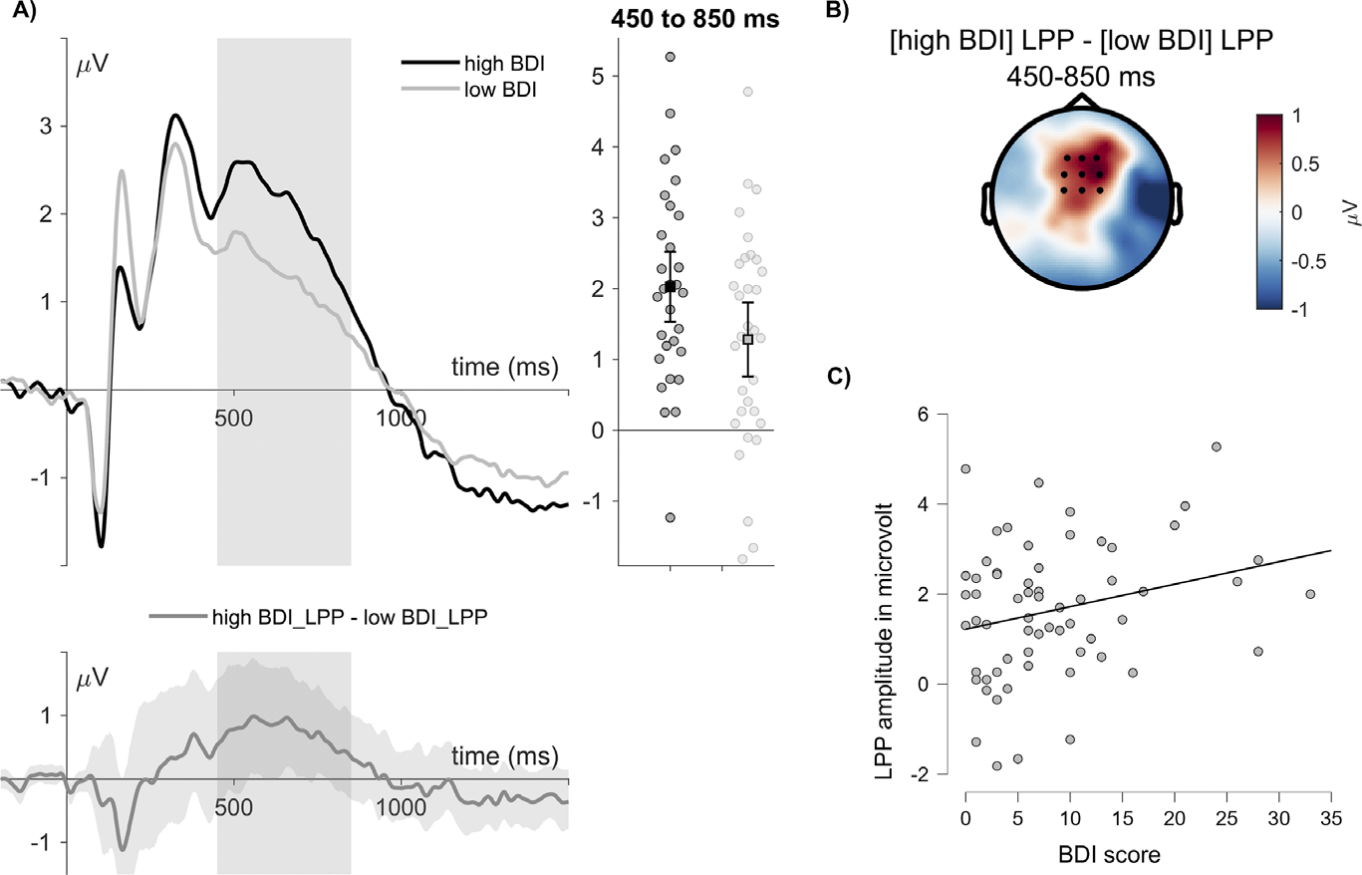
LPP correlations between BDI scores and LPP amplitudes. (a) ERP waveforms show the time course for the median split (BDI < 6, *N* = 32; BDI > 6, *N* = 30), selected for display purposes. Error bars show 95% confidence intervals. Difference plots contain 95% bootstrap confidence intervals of intra-individual differences. (b) The scalp topography depict the amplitude differences between participants with high and low BDI scores. (c) Scatterplot showing the correlation between the BDI scores and LPP amplitudes.

We did not observe BDI relationships with LPP amplitude differences between worse and better feedback (Pearson *r* = −.056, *p =* .663), but a significant correlation between BDI scores and the collapsed LPP, showing that participants with larger BDI scores exhibited a larger LPP to all feedback (Pearson *r* = .255, *p =* .045; see Figure 4).

## Discussion

We tested neuronal and behavioral responses toward social evaluative feedback, examining the integration of the feedback into the self-view and simultaneous changes in feedback expectations based on the sender’s behavior. We varied sender characteristics (attributed expertise and manipulated behavior) and receiver characteristics (selected variability of depression scores). We found that feedback expectation changed according to the sender’s attitude over time, with participants expecting more negative feedback from negative senders. This effect was pronounced in participants with higher depression scores, even when controlling for their more negative initial self-view. Regarding self-view updating, participants generally exhibited a larger magnitude of change following better feedback and thus showed a positively biased updating. Overall, participants with higher BDI scores showed a more negative self-view updating, while BDI scores selectively correlated with worse feedback from the experts and better feedback from the peers. Concerning ERPs toward the feedback, the incongruence of the feedback with the expectation, but not with the self-view, increased FRN amplitudes. In contrast, the LPP was increased for both types of incongruence, and LPP amplitudes were also related to the BDI scores in general, with higher LPP values.

Concerning feedback expectations, we observed significant differences across the experiment that became statistically significant after 10–18 trials, dissociating negative and positive senders, while overall feedback expectations were more negative than the self-ratings. This effect was pronounced in participants with higher BDI scores, even though their initial self-ratings were more negative than for those with lower scores. In general, changes in feedback expectations were not positively biased and, further, not equally for participants with higher BDI scores concerning positive and negative information (Strunk et al., 2006; Sharot, 2011). In the predictive processing model of depression, cognitive immunization processes are reasoned to lead depressed participants to maintain negative expectations when facing unexpected positive information but adapt to unexpected negative information (Kube et al., 2020). Another view on the changes of expectations is that feedback ratios were manipulated so that ‘negative’ senders, on average, gave feedback that was one point more negative than the self-ratings, which were accurately predicted in participants with high BDI values (see Figure 1d). However, we observe a dissociation in expectation and self-view changes (see below).

Concerning the impact of social evaluative feedback on self-view changes, we found a significant effect of feedback congruence with the self-view and an interaction between sender expertise and feedback congruence. Participants did not change their self-ratings substantially after receiving feedback congruent with the self-rating but showed a stronger change in magnitude after better than after worse feedback relative to the self-rating. Interestingly, the interaction showed that participants updated more negatively when the expert provided worse feedback than the initial self-evaluation. Importantly, the self-view updating was generally correlated with self-reported depression symptoms. Follow-up correlations related this most strongly to more negative updating after negative expert feedback and a lack of positive updating for positive peer feedback. Our findings are partly in line with theoretical viewpoints of a general positive bias of integrating self-relevant new information (Sharot et al., 2011; Sharot & Garrett, 2016) and asymmetrical self-view updating to more positive ratings (Elder et al., 2022; Korn et al., 2012). Previous behavioral findings also indicated that attributed sender status helps to integrate and adapt feedback being worse than one’s self-view; namely, negative feedback from high-status experts (e.g. senior therapists) was more accepted than from low-status experts (Collins & Stukas, 2006). Interestingly, we observe that this effect is specifically increased in participants with higher self-reported depressive symptoms. As participants with high BDI scores also show a lack of positive updating for peer feedback, this may specify the predictive processing model by Kube and colleagues concerning depression (Kube et al., 2020; see above). More recently, Kube suggested that the integration of new positive information is distorted in depressed participants, which we found to be the case for peer but not expert feedback (Kube, 2023).

Concerning the ERP modulations in response to the social evaluative feedback congruence to the self-view and the feedback expectation, we examined the FRN and LPP components, indexing responses toward different types of incongruence (FRN) and late processes of feedback evaluation and integration (LPP). Concerning the FRN, no modulations of the FRN amplitudes to the feedback congruence with the self-view but with the feedback expectation were observed. Unexpected feedback led to increased FRN amplitudes, similar to accounts that propose that FRN amplitudes reflect an unsigned prediction error (Walentowska, Severo, Moors, & Pourtois, 2019), also more reliably shown in the domain of social evaluative feedback studies (Peters et al., 2024). A different view emerged on the role of this component (Becker et al., 2014; Foti, Weinberg, Dien, & Hajcak, 2011; Holroyd, Pakzad-Vaezi, & Krigolson, 2008), describing that this component reflects the variability of positive deflections to positive outcomes (Bernat et al., 2015; Foti et al., 2015), also reported to correlate with depressive symptoms in specific social evaluative feedback designs (Pegg et al., 2019, 2021). However, we could not observe a specific relationship of amplitudes toward better or other feedback types with ERP amplitudes, nor did we observe an absent difference between better and worse feedback in the LPP (see Xie et al., 2022). We explored a possible relationship between LPP amplitudes and BDI scores, showing a generally increased LPP response toward all feedback. The LPP was sensitive to both the incongruence of feedback with the self-view and with the feedback expectation. This pattern agrees with previous research that shows that incongruent feedback elicits higher LPP amplitudes (e.g. see Schindler et al., 2021). Here, we observed no main effects of the sender, while an interaction showed that expert feedback did not differ when considering the feedback congruence with the self-view (see Schindler et al., 2019). Correlations between BDI scores and overall LPP amplitude may be viewed as a more elaborate stimulus processing, evaluation, and encoding process in participants with higher depression scores (Dolcos & Cabeza, 2002; Hajcak et al., 2010; Schindler, Vormbrock, & Kissler, 2022; Schupp et al., 2006).

### Constraints of generality and outlook

A limitation to consider is that the feedback deviation from participants’ self-view was manipulated, with senders having intermixed different proportions of better, congruent, and worse feedback trials. While this maintains ecological credibility, it reduces the ability to predict feedback. Further, the subtle self-incongruent feedback (i.e. one point deviating) may not have elicited effects as strongly as reported in other studies that found relationships between depression and ERP response. We may have missed possible specific relationships with individual depressive symptoms. Future studies need to implement high ecological validity and simultaneously enable participants to form clear expectations and differentiate between negative and positive feedback. The current subclinical sample, which is primarily comprised of young, highly educated female students, limits the generalizability of the findings to the broader population. Future research should examine the potential impact of possible influences of gender, the constrained age range, and possible differences in anxiety levels on the processing of social evaluative feedback. Notably, given the known gender differences in emotional processing, specifically on ERP components such as the LPP (e.g. see Schirmer & McGlone, 2019), the gender imbalance in the sample may have impacted the observed effects. We performed control analyses that included gender as a between-subjects factor (see Supplementary Sections 1–3), showing no impact of gender on statistical results, except for feedback expectation ratings, where one interaction between sender attitude and block became insignificant. Still, due to the small sample size, we might underestimate the influence of gender on ERP differences, and therefore, future studies should sample balanced gender samples to more broadly generalize the obtained results.

## Conclusion

This study provides insights into the effects of social evaluative feedback on updating processes of the self-view and the feedback expectations, emphasizing the roles of sender and receiver characteristics. As outlined by Beck (1979), participants with high BDI scores exhibited more negative views of self (t1 ratings, self-view update), future, and world (feedback expectations). On the other hand, for the whole sample, we see the overall positivity bias in self-view updating, seeming dissociated from feedback expectations, with participants expecting rather similar or even more negative feedback but updating selectively for feedback being more positive than the self-rating. BDI scores were correlated with the LPP amplitude to all feedback. These results contribute to a deeper understanding of how individuals process and integrate social evaluative feedback regarding self-views and feedback expectation updates and how these correlate with depressive symptoms at the behavioral level.

## Supporting information

Helming et al. supplementary material Helming et al. supplementary material

## Supplementary material

The supplementary material for this article can be found at http://doi.org/10.1017/S0033291725102511.
