# Supplementary material for: Effects of depressive symptoms on neuronal processing of social evaluative feedback and subsequent changes in expectations and self-view
Source: Psychol Med. 2025 Nov 26;55:e361. doi: 10.1017/S0033291725102511 (PMC12671916; doi:10.1017/S0033291725102511)
Supplement: Helming et al. supplementary material [file S0033291725102511sup001.docx]

**Effects of depressive symptoms on neuronal processing of social evaluative feedback and subsequent changes in expectations and self-view**

**SUPPLEMENTARY MATERIALS**

Hanne Helming^1*^, Antje Peters^1,2^, Franka Hüttenhein^1^, Robert Moeck^1^, Thomas Straube^1,2^, and Sebastian Schindler^1,2^

^1^Institute for Medical Psychology and Systems Neuroscience, University of Münster, Germany

^2^ Otto Creutzfeldt Center for Cognitive and Behavioral Neuroscience, University of Muenster

* Corresponding author

**Correspondence addresses**

University of Münster

Institute of Medical Psychology and Systems Neuroscience

Von-Esmarch Straße 52, 48149 Münster

E-mail: [hanne.helming@uni-muenster.de](mailto:hanne.helming@uni-muenster.de)

Analyses with the between-subjects factor of gender

1. **Methods**

***Feedback Expectation***

For behavioral effects, we tested feedback expectation ratings over time and self-view updating according to the feedback type and sender behavior. For expectation ratings, we averaged the expectation values within each trial across participants and five consecutive blocks, each containing 9 trials per sender. We calculated a repeated measures ANOVA with the within factors sender expertise (two levels: expert vs. peer), sender attitude (two levels: negative vs. positive), and block (five levels: 'Block 1', 'Block 2', 'Block 3', 'Block 4', and 'Block 5') and the between-subjects factor gender.

***Self-View***

Concerning self-view updates, the update values were calculated based on differences between the initial rating and the re-evaluation after receiving feedback (t2 r' – t1 r'), where negative values index negative changes and positive values index positive changes. Here, a Repeated Measures ANOVA with the within factors sender expertise (two levels: 'peer' vs. 'expert') and feedback self-view congruence (three levels: 'better', 'congruent', 'worse'), as well as the between-subjects factor gender, was calculated.

***ERP analyses***

We performed a Repeated Measures ANOVA with the within factors of sender expertise (two levels: 'expert' vs. 'peer') and feedback congruence to the self-view (three levels: 'better', 'congruent', 'worse') for FRN and LPP mean amplitudes, and the between subjects factor gender. Additionally, we run ANOVAs using the participants' feedback prediction with the within factors of sender expertise (two levels: 'expert' vs. 'peer') and feedback expectedness (three levels: 'better', 'expected', 'worse'), along with the between-subjects factor gender.

1. **Results**

Please note that all analyses that differ in reported significance from those reported in the main document are highlighted in blue font size.

- 1. **Behavioral Results**

***Feedback Expectation***

The ANOVA with the between subjects factor gender revealed no main effect of sender expertise (*F*_(1,61)_ = 0.58, *p* = .448, η_P_² < .001) and no interaction of sender expertise and gender (*F*_(1,61)_ = 0.18, *p* = .676, η_P_² < .001), no main effect of block (*F*_(4,240)*_ = 0.59, *p* =.674, η_P_² = .001) and no interaction with gender (*F*_(4,240)*_ = 0.91, *p* =.461, η_P_² = .002), a main effect of sender attitude (*F*_(1,61)_ = 58.39, *p* < .001, η_P_² = .051) but no interaction with gender (*F*_(1,61)_ = .472, *p* = .495, η_P_² < .001). Post-hoc analyses showed that for the main effect of sender attitude, feedback expectations were more positive for the positive senders than for the negative senders (t = 7.64, *p*_holm_ < .001, Cohen's d = 0.505). There was no interaction of sender expertise and sender attitude (*F*_(1,60)_ = 0.02, *p* = .900, η_P_² < .001), sender expertise and block (*F*_(4,240)_ = 0.72, *p* = .580, η_P_² = .001), sender attitude and block (*F*_(4,240)_ = 2.38, *p* = .053, η_P_² = .005) and no three-way interaction (*F*_(4,240)_ = 1.05, *p* = .384, η_P_² = .002). Given the change in the interaction between sender attitude and block, we explore the effects for female and male participants separately. For females, positive and negative senders did not differ significantly in the first block (*t* = 2.26, *p*_holm_ = .520, Cohen's d = 0.309), and in the second block (*t* = 2.28, *p*_holm_ = .513, Cohen's d = 0.307), they did so in the third (*t* = 3.26, *p*_holm_ = .036, Cohen's d = 0.445), fourth (*t* = 5.38, *p*_holm_ < .001, Cohen's d = 0.735), and fifth block (*t* = 5.99, *p*_holm_ < .001, Cohen's d = 0.820). For males, positive and negative senders did not differ significantly in the first (*t* = 0.65, *p*_holm_ = 1.00, Cohen's d = 0.142), in the second (*t* = 2.87, *p*_holm_ = .192, Cohen's d = 0.627), in the third (*t* = 2.43, *p*_holm_ = .593, Cohen's d = 0.532), fourth (*t* = 2.18, *p*_holm_ 1.00, Cohen's d = 0.477), or the fifth block (*t* = 2.92, *p*_holm_ = .171, Cohen's d = 0.640). Further there were no three-way interactions of sender expertise x sender attitude x gender (*F*_(4,240)_ = 0.28, *p* = .596, η_P_² < .001), sender expertise x block x gender (*F*_(4,240)_ = 1.53, *p* = .195, η_P_² = .003), sender attitude x block x gender (*F*_(4,240)_ = .767, *p* = .584, η_P_² = .002), and sender expertise x sender attitude x block x gender (*F*_(4,240)_ = 2.38, *p* = .052, η_P_² = .006). Furthermore, there was no main effect of gender (*F*_(1,61)_ = 2.63, *p* = .110, η_P_² = .012).

***Self-View***

The ANOVA with between factor gender revealed no main effect of sender expertise (*F*_(1,61)_ = 1.49, *p* = .227, η_P_² < .001) and no interaction with gender (*F*_(1,61)_ = 0.80, *p* = .375, η_P_² < .001), a main effect of feedback congruence with the self-view (*F*_(2,120)_ = 186.90, *p* < .001, η_P_² = .510) and an interaction with gender (*F*_(2,120)*_ = 5.29, *p* = .006, η_P_² = .014). There was an interaction of sender expertise and feedback congruence (*F*_(2,120)_ = 4.47, *p* = .013, η_P_² = .004), but no three-way interaction with gender (*F*_(2,120)_ = 1.48, *p* = .231, η_P_² = .001). Post-hoc analyses showed for the main effect of feedback congruence with the self-view that participants exhibited a significantly more positive self-view updating after receiving better (*t*_(59)_ = 18.63, *p*_holm_ < .001, Cohen's d = 2.625) or congruent feedback (*t*_(59)_ = 4.82, *p*_holm_ < .001, Cohen's d = 0.680) compared to worse feedback. Furthermore, better feedback also led to significantly more positive updating compared to congruent feedback (*t*_(59)_ = 13.80, *p*_holm_ < .001, Cohen's d = 1.95). Concerning the interaction of feedback congruence with the self-view and gender, updating did not differ between gender after congruent feedback (*t*_(59)_ = 0.58, *p*_holm_ = .147, Cohen's d = 0.560), and after better feedback than the self-view(*t*_(59)_ = 1.68, *p*_holm_ = .276, Cohen's d = 0.427), while males engaged in more negative updating after worse feedback than the self-view(*t*_(59)_ = -1.90, *p*_holm_ = .236, Cohen's d = -0.479). Concerning the interaction of sender expertise and feedback congruence with the self-view, updating did not differ between the senders after congruent feedback (*t*_(59)_ = 0.79, *p*_holm_ = .864, Cohen's d = 0.088), and after better feedback than the self-view(*t*_(59)_ = 0.23, *p*_holm_ = .864, Cohen's d = 0.025), while the expert sender led to more negative updating after worse feedback than the self-view(*t*_(59)_ = -3.13, *p*_holm_ = .008, Cohen's d = -0.350). Furthermore, there was no main effect of gender (*F*_(1,61)_ = 0.12, *p* = .731, η_P_² < .001).

- 1. **EEG Results**

***FRN self-view***

The ANOVA with the between subjects factor gender revealed no main effect of sender expertise (*F*_(1,61)_ = 0.02, *p* = .899, η_P_² < .001) and no interaction with gender (*F*_(1,61)_ < .001, *p* = .993, η_P_² < .001), no main effect of self-view congruence (*F*_(2,122)_ = 1.77, *p* = .174, η_P_² = .002) and no interaction with gender (*F*_(2,122)_ = 0.15, *p* = .859, η_P_² < .001). There was no interaction between sender expertise and self-view congruence (*F*_(2,122)*_ = 0.03, *p* = .975, η_P_² < .001; see Supplementary Figure S1), and no three-way interaction with gender (*F*_(2,122)*_ = 1.79, *p* = .172, η_P_² = .001). Furthermore, there was no main effect of gender (*F*_(1,61)_ = 2.06, *p* = .157, η_P_² = .029).

**Supplementary Figure S1. FRN effects of feedback incongruence with the initial self-view ratings for A) male and B) female participants.** ERP waveforms show the time course for worse (red/pink), congruent (dark/light grey), and better feedback (dark/light blue lines) for the 'peer' and 'expert' senders. Error bars show 95% confidence intervals. Difference plots contain 95% bootstrap confidence intervals of intra-individual differences. The scalp topographies below depict the amplitude differences for the worse/better feedback and the congruent/expected feedback.


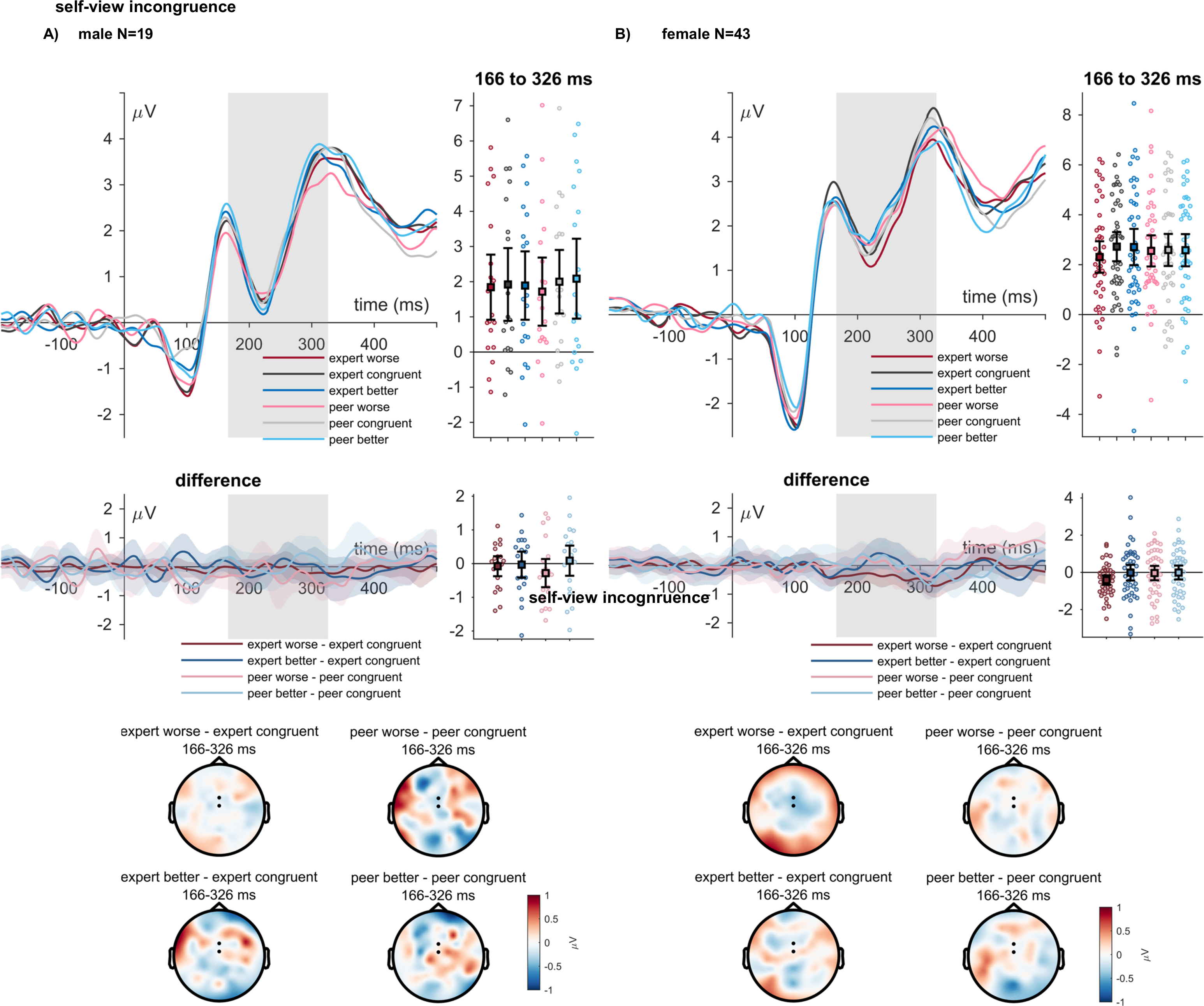


***FRN expectation***

The ANOVA with the between-subjects factor gender revealed no main effect of sender expertise (*F*_(1,61)_ = 1.59, *p* = .212, η_P_² = .001; see Supplementary Figure S2) and no interaction with gender (*F*_(1,61)_ = 2.34, *p* = .131, η_P_² = .001). There was a main effect of the expectation (*F*_(2,122)*_ = 5.92, *p* = .004, η_P_² = .010), but no interaction with

**Supplementary Figure S2. FRN effects of feedback incongruence with the expectation ratings in the current trial for A) male and B) female participants.** ERP waveforms show the time course for worse (red/pink), congruent (dark/light grey), and better feedback (dark/light blue lines) for the 'peer' and 'expert' senders. Error bars show 95% confidence intervals. Difference plots contain 95% bootstrap confidence intervals of intra-individual differences. The scalp topographies below depict the amplitude differences for the worse/better feedback and the congruent/expected feedback.


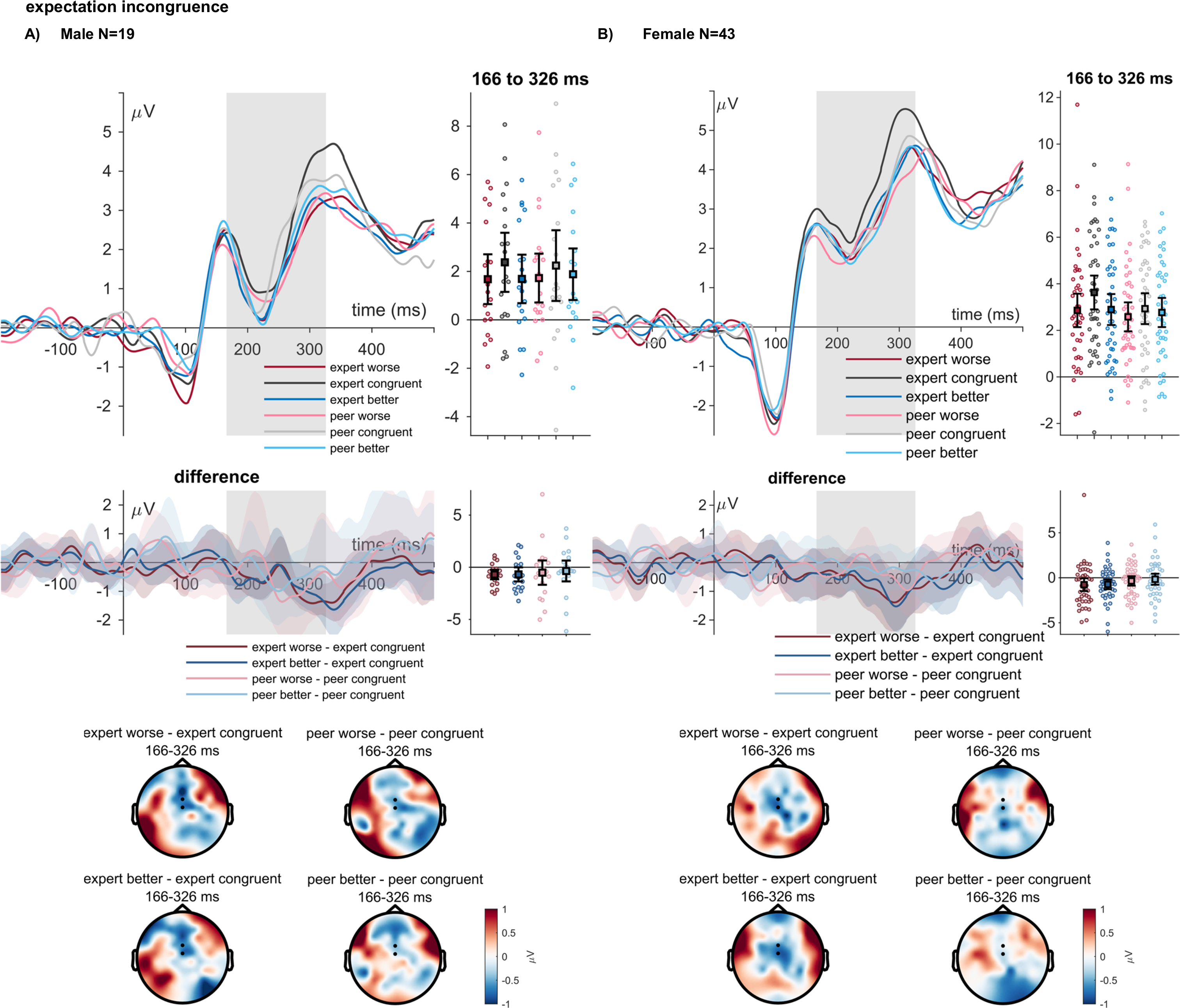


gender (*F*_(2,122)*_ = 0.02, *p* = .979, η_P_² < .001). There was no interaction between sender expertise and expectation (*F*_(2,122)_ = 0.73, *p* = .486, η_P_² = .001) and no interaction with gender (*F*_(2,122)*_ = 0.07, *p* = .936, η_P_² < .001). Post-hoc tests showed a significantly increased FRN for worse feedback than for expected feedback (*t*_(61)_ = -3.22, *p*_holm_ = .005, Cohen's d = -.245) and for better feedback than for expected feedback (*t*_(61)_ = - 2.67, *p*_holm_ = .017, Cohen's d = -.204). There was no significant difference between worse and better feedback (*t*_(61)_ = -0.54, *p*_holm_ = .588, Cohen's d = -.041). Furthermore, there was no main effect of gender (*F*_(1,61)_ = 2.18, *p* = .080, η_P_² = .038).***LPP self-view***

The ANOVA with the between subjects factor gender revealed no main effect of sender expertise (*F*_(1,61)_ = 0.12, *p* = .731, η_P_² < .001; see Supplementary Figure S3) and no interaction of sender expertise and gender (*F*_(1,61)_ < 0.01, *p* = .959, η_P_² < .001), a main effect of feedback congruence with the self-view (*F*_(2,120)_ = 11.24, *p* < .001, η_P_² = .013) but no interaction of feedback congruence with the self-view and gender (*F*_(2,120)_ = 0.25, *p* = .781, η_P_² < .001). There was an interaction of sender expertise and feedback congruence (*F*_(2,120)*_ = 5.550, *p* = .005, η_P_² = .005) but no three-way interaction with gender (*F*_(2,120)*_ = .526, *p* = .592, η_P_² < .001). Post-hoc analyses showed for the main effect of feedback congruence with the self-view that participants exhibited a significantly more positive self-view updating after receiving better (*t*_(59)_ = 2.01, *p*_holm_ = .047, Cohen's d = 0.134) or congruent feedback (*t*_(59)_ = 4.72, *p*_holm_ < .001, Cohen's d = 0.316) compared to worse feedback. Furthermore, better feedback also led to significantly more positive updating compared to congruent feedback (*t*_(59)_ = -2.72, *p*_holm_ =.015, Cohen's d = -0.182). Concerning the interaction of sender expertise and feedback congruence with the self-view, LPP amplitudes did not differ between the senders after congruent feedback (*t*_(59)_ = 2.39, *p*_holm_ = .161, Cohen's d = 0.221), better feedback than the self-view (*t*_(59)_ = 0.11, *p*_holm_ = 1.00, Cohen's d = 0.010), and also not after worse feedback than the self-view (*t*_(59)_ = 1.79, *p*_holm_ = .531, Cohen's d = 0.165). Furthermore, the main effect of gender was significant (*F*_(1,61)_ = 8.07, *p* = .006, η_P_² = .097).

**Supplementary Figure S3. LPP effects of feedback incongruence with initial self-view ratings for A) male and B) female participants.** ERP waveforms show the time course for worse (red/pink), congruent (dark/light grey), and better feedback (dark/light blue lines) for the 'peer' and 'expert' senders. Error bars show 95% confidence intervals. Difference plots contain 95% bootstrap confidence intervals of intra-individual differences. The scalp topographies below depict the amplitude differences for the worse/better feedback and the congruent/expected feedback.


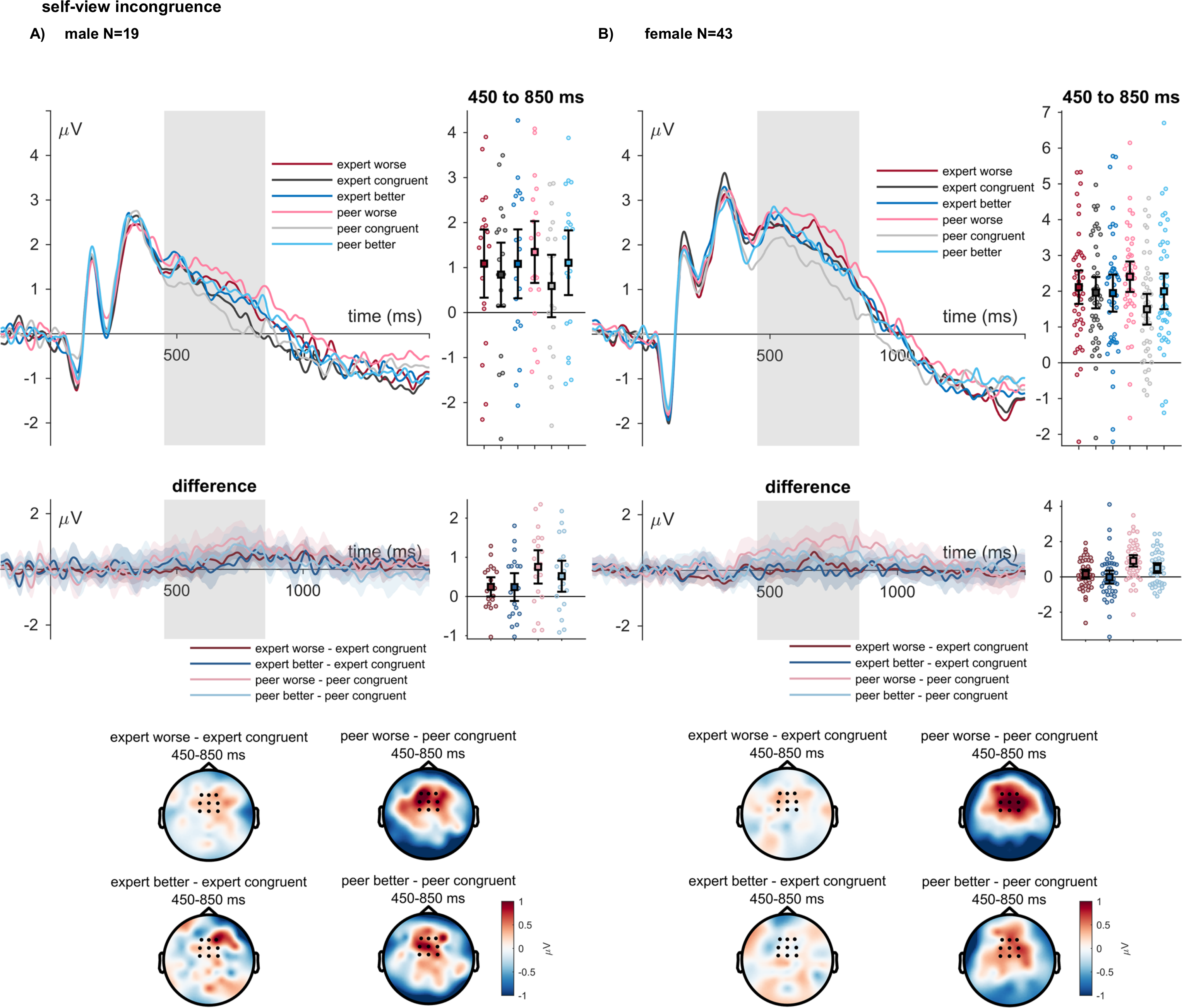


***LPP expectation***

The ANOVA with the between subjects factor gender revealed no main effect of the sender expertise (*F*_(1,61)_ = 1.37, *p* = .246, η_P_² = .001) and no interaction with gender (*F*_(1,61)_ < .001, *p* = .994, η_P_² < .001), a main effect of expectation (*F*_(2,122)*_ = 5.35, *p* = .006, η_P_² = .009) but no interaction with gender (*F*_(2,122)*_ = 0.09, *p* = .911, η_P_² < .001), and no interaction between sender expertise and expectation (*F*_(2,122)*_ = 1.95, *p* = .146, η_P_² = .004; see Supplementary Figure S4) and no three-way

**Supplementary Figure S4. LPP effects of feedback incongruence with expectation ratings in the current trial for A) male and B) female participants.** ERP waveforms show the time course for worse (red/pink), congruent (dark/light grey), and better feedback (dark/light blue lines) for the 'peer' and 'expert' senders. Error bars show 95% confidence intervals. Difference plots contain 95% bootstrap confidence intervals of intra-individual differences. The scalp topographies below depict the amplitude differences for the worse/better feedback and the congruent/expected feedback.


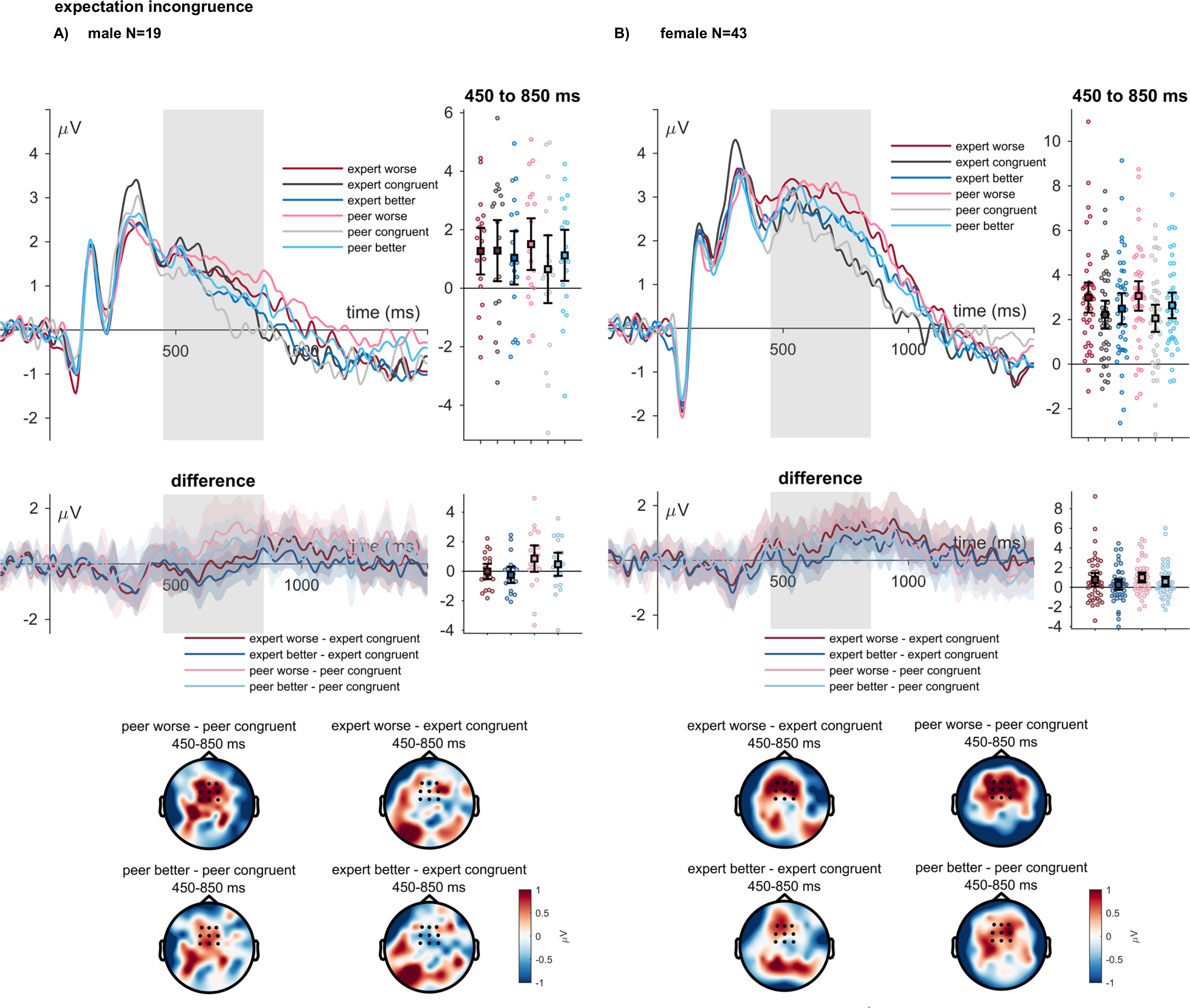


interaction with gender (*F*_(2,122)*_ = 0.10, *p* = .908, η_P_² < .001). Post-hoc tests showed a significantly increased LPP for worse feedback than for expected feedback (*t*_(61)_ = -3.27, *p*_holm_ = .004, Cohen's d = -0.256) and for better feedback than for expected feedback (*t*_(61)_ = - 1.71, *p*_holm_ = .181, Cohen's d = -0.134). There was no significant difference between worse and better feedback (*t*_(61)_ = 1.56, *p*_holm_ = .181, Cohen's d = 0.122). Furthermore, there was a main effect of gender (*F*_(1,61)_ = 9.28, *p* = .003, η_P_² = .095).

1. **Discussion**

Taking gender into account as a between-subjects factor, the results show the same effects as those without accounting for gender. Only the interaction of sender attitude and block became non-significant concerning feedback expectations (*F*_(4,240)_ = 2.38, *p* = .053, η_P_² = .005). However, directed post-hoc comparisons here show that for both females and males, differences increase after the first to the fifth block. Due to the multiple comparison correction and lower statistical power of explorations within the smaller female and male samples separately, we found that differences in feedback expectations between positive and negative senders became significant only starting with the third within the large sample of females. However, in males, due to the even smaller sample, these differences did not reach corrected significance thresholds across the full five blocks. Regarding the ERP analyses, there are some general differences between male and female participants, with higher positivity in females observed both during the FRN and LPP amplitudes. For the more important differential effects, we could not find interactions between gender and the main effects of sender, feedback congruence, or between sender and feedback congruence. However, the sample is small and unbalanced, with only nineteen males compared to forty-three females. Thus, we lack the statistical power to quantify the absence of differential processing, and future studies require larger, more balanced samples to address this issue. Nevertheless, for a complete picture, we include the visualization of possible and more descriptive differences in the Supplementary Figures S1-S4.
